# Qualitative Behavioral Assessment in Juvenile Farmed Atlantic Salmon (*Salmo salar*): Potential for On-Farm Welfare Assessment

**DOI:** 10.3389/fvets.2021.702783

**Published:** 2021-09-07

**Authors:** Susan Jarvis, Maureen A. Ellis, James F. Turnbull, Sonia Rey Planellas, Francoise Wemelsfelder

**Affiliations:** ^1^The Global Academy of Agriculture and Food Security, University of Edinburgh, Edinburgh, United Kingdom; ^2^Institute of Aquaculture, Faculty of Natural Sciences, University of Stirling, Stirling, United Kingdom; ^3^Animal and Veterinary Sciences, SRUC (Scotland's Rural College), Edinburgh, United Kingdom

**Keywords:** qualitative behavioral assessment, fish, salmon, aquaculture, welfare

## Abstract

There is a growing scientific and legislative consensus that fish are sentient, and therefore have the capacity to experience pain and suffering. The assessment of the welfare of farmed fish is challenging due to the aquatic environment and the number of animals housed together. However, with increasing global production and intensification of aquaculture comes greater impetus for developing effective tools which are suitable for the aquatic environment to assess the emotional experience and welfare of farmed fish. This study therefore aimed to investigate the use of Qualitative Behavioral Assessment (QBA), originally developed for terrestrial farmed animals, in farmed salmon and evaluate its potential for use as a welfare monitoring tool. QBA is a “whole animal” approach based on the description and quantification of the expressive qualities of an animal's dynamic style of behaving, using descriptors such as relaxed, agitated, lethargic, or confident. A list of 20 qualitative descriptors was generated by fish farmers after viewing video-footage showing behavior expressions representative of the full repertoire of salmon in this context. A separate, non-experienced group of 10 observers subsequently watched 25 video clips of farmed salmon, and scored the 20 descriptors for each clip using a Visual Analog Scale (VAS). To assess intra-observer reliability each observer viewed the same 25 video clips twice, in two sessions 10 days apart, with the second clip set presented in a different order. The observers were unaware that the two sets of video clips were identical. Data were analyzed using Principal Component (PC) Analysis (correlation matrix, no rotation), revealing four dimensions that together explained 79% of the variation between video clips, with PC1 (Tense/anxious/skittish—Calm/mellow/relaxed) explaining the greatest percentage of variation (56%). PC1 was the only dimension to show acceptable inter- and intra-observer reliability, and mean PC1 scores correlated significantly to durations of slow and erratic physical movements measured for the same 25 video clips. Further refinements to the methodology may be necessary, but this study is the first to provide evidence for the potential of Qualitative Behavioral Assessment to serve as a time-efficient welfare assessment tool for juvenile salmon under farmed conditions.

## Introduction

While global fish supply from capture has remained relatively static since the mid 1980's, there has been a huge increase in both inland and marine aquaculture (1) with much of this increase in Asia, and China in particular. In 2018, it is estimated that 54.3 million tons of finfish were produced globally within aquaculture with salmon accounting for 4.5% of global production (1). In Scotland alone it is estimated that 47 million fish were transferred from freshwater rearing tanks to sea cages in 2018 (2). Farmed fish can be held in different types of rearing system, and are subject to varying husbandry routines and operations throughout the different stages of their life cycle. These systems will impact fish welfare in different ways, exposing them to different stress challenges and hazards, and presenting the risk that the animals' environmental and behavioral needs, both at individual and group level, are not met (3). Given the rapid increase in aquaculture production and the range of species now farmed, there is thus an urgent need to address the welfare of farmed fish, and, as with other farmed animal species, to develop methods that monitor the different species' needs (4).

Welfare appraisal in fish has frequently focused on disruption of biological function, illness, injury and mortality. However, a “feelings-based” consideration of animal welfare (5) has historically been neglected in fish welfare assessment, along with consideration of opportunities for positive affect and well-being (6, 7). For example, in Scotland, welfare inspection and enforcement, outside of assurance scheme requirements, is under the remit of local authorities, government and Animal Health and Veterinary Laboratories Agency. These inspections are often carried out in response to reports of mass disease or mortality (6) and in such cases, assessment is often primarily concerned with mortality, clinical indicators of disease and inappropriate usage of veterinary medicines (6).

Assurance scheme welfare guidance frameworks, including the Code of Good Practice for Finfish Aquaculture (8) and RSPCA Assured welfare standards for Atlantic salmon and rainbow trout (9), are based on the “Five Freedoms,” which mostly focus on the avoidance of negative states such as pain and hunger. The importance of positive experiences for welfare are receiving growing recognition (10, 11), however suitable methodologies to robustly assess positive welfare in different species are still lacking and in need of development (12).

There is a growing body of evidence supporting that fish are intelligent, sentient beings that possess cognitive abilities of considerable complexity [e.g., (13, 14)], and are capable of emotion and experiencing pain (15, 16). These capacities do not in fact appear far removed from those observed among warm-blooded terrestrial vertebrates, yet the level of protection and moral concern afforded to fish remains far behind that given to terrestrial species (15, 17). For example, in the European Council Directive 98/58/EC^1^ on the protection of animals for farming purposes, the detailed welfare provisions prescribed exclude fish (6), despite the explicit acknowledgment of fish sentience in EU law^2^. Fish species have been afforded greater protection when used for experimental purposes however, through national legislations, regulations and guidelines [e.g., (18, 19)]. In Europe^3^ fish in scientific settings are protected from the time they are capable of independent feeding, on the assumption they are then capable of experiencing pain, suffering and distress. Following this directive, the UK Animals Scientific Procedures Act 1986^4^ included fish for the first time as worthy of protection, but this does not apply to fish in commercial aquaculture.

Current monitoring methods within the aquaculture industry are limited to video surveillance measures of the physical environment such as water turbidity, however there is scope to expand the use of such technology to include a wider range of welfare indicators (20–22). Stien et al. (23) review existing welfare standards and assessment systems for farmed fish and suggest a system of Operational Welfare Indicators that can be monitored through video surveillance, as practiced for example in Norway. An additional problem in operating such systems, however, is that a progressive decrease of staff relative to fish numbers imposes time constraints on monitoring. Recent reviews of Scottish aquaculture have shown that tonnage of seawater fish produced in relation to number of employees has increased by 11-fold since 1985 (24). There is thus a distinct requirement for time-efficient fish welfare assessment tools which, as is increasingly the case with terrestrial animals, should not only focus on physical well-being, but also on emotional well-being, including both negative and positive experiences (11).

Qualitative Behavioral Assessment is an integrative technique which evaluates the “whole animal” in terms of the dynamic expressive quality of its behavior (25, 26). Different “styles” of behavior are summarized using qualitative descriptors such as relaxed, agitated, inquisitive and listless (27) that should cover the full range of both negative and positive emotional experience, and are quantified by scoring their prevalence on unstructured Visual Analog Scales. Numerous studies have validated the application of QBA to different livestock species (28). A perceived strong point of QBA is that it includes positive aspects of animal affect, which led to its inclusion in EU Welfare Quality® welfare assessment protocols for cattle, pigs and poultry as the only indicator for positive emotional state [e.g., for poultry: (29)]. In addition, integrative judgements of expressivity are time efficient (30), and so potentially provide a logistically feasible tool for practical on-farm welfare assessments. However, integrative judgments also bring vulnerabilities; people are known to vary in the way they calibrate unstructured Visual Analog Scales, potentially confounding outcomes with undesirable observer-based variation (26). It is therefore best to always use QBA in combination with other validated animal- and resource-based measures, and adequate instruction and training are essential (31).

To date, QBA has not been applied to fish. Whilst in terrestrial species observers may integrate expressive qualities of elements such as posture (32), facial expression (33) and ear position (34) into assessments of overall body language, there may be fewer such elements available for assessment in fish. The scientific literature reports a range of health and welfare measures for fish, including behaviors such as food intake, swimming and stereotyped behavior (23, 35). However, there is a lack of measures for affective state such as facial expression (36), and also of efforts to integrate different measures of behavior into assessments of affective state. A challenge for assessing fish affect is the number of fish that are kept within sea cages and the aquatic environment, limiting their movement and expressivity. However, QBA has been applied successfully to large groups of terrestrial animals such as commercial broilers (37, 38), suggesting that potentially this is also possible for fish.

The aim of this study was to evaluate the inter- and intra- observer reliability of observer judgements of fish body language using a fixed list QBA methodology developed for juvenile Atlantic salmon. In addition, the association between QBA scores and measurements of ethogram-based categories of salmon behavior was investigated for the purpose of additional validation.

## Materials and Methods

### Ethical Review

Approval for video recording was gained from the University of Stirling Animal Welfare & Ethical Review Body and SRUC's Animal Ethical Committee. Approval for observer participation in the QBA element was gained from the University of Edinburgh Vet School Human Ethical Research Committee (HERC: approval number HERC_79_17).

### Animals, Housing, and Husbandry

The fish used in this study were juvenile Atlantic salmon (Salmo salar), kept at a hatchery and rearing unit in Scourie, Scotland between December and April 2017. The fish were 9–12 months of age and weighed around 30–45 g. The unit contained 23 freshwater circular rearing tanks (5 m diameter by 2 m deep) in a flow-through land-based system with no artificial current. There were ~13,000 juvenile salmon in each freshwater tank and stocking density was on average 20 Kg/m^3^. The 23 tanks were laid out in 5 rows of 4 with a row of 3 at one end, and the enrichment was spread throughout the layout.

One tank was not populated by salmon at any point in the study. Three of the tanks were excluded due to poor visibility in the water. Of the remaining 19 populated tanks in which salmon were assessed, 9 were randomly assigned to contain environmental enrichment in the form of artificial “kelp” which was suspended from above the water in the form of long plastic strips. The other 10 tanks did not contain artificial kelp but were otherwise identical in setup. The sex ratio of fish was unknown, as during the pre-smoltification stage, the salmon had not reached sexual maturity and displayed no external indicators of gender. All salmon in the rearing tanks were fed on standard salmon pelleted dry food (Skretting Nutra Advance/Supreme^©^), which was deployed by automatic feeders using a spinning arm every 10–20 min during daylight hours. Fish were routinely vaccinated at 7 months of age. Daily tank cleaning was carried out between 0900 and 1100 h (aside from on video recording days, where this was performed after each tank was recorded). During this process any salmon mortalities were removed.

### Video Recording

Video recording was carried out in the 19 tanks populated with salmon with submerged GoPro Hero 3^©^ cameras. That this included tanks both with and without enrichment was not considered a problem, as this difference could be expected to increase the range of salmon expressivity available for observers to assess. During the recording of each tank, the GoPro^©^ cameras were submerged using a metal pole which was fixed in place for 17 min (the length of one undivided “block” of footage as recorded by the camera). The GoPro's field of view normally showed between 10 and 40 fish. All recordings were carried out between 10:00 and 13:00 h. During all recording periods, workers were instructed not to perform any husbandry procedures or use the walkways over the tanks during recording so as not to disturb the fish. In accordance with the standard feeding routine, the automatic feeders continued to deploy food every 20 min.

### Qualitative Behavioral Assessment

Qualitative Behavioral Assessment was carried out in two phases. Phase 1 consisted of the generation of a list of terms for describing salmon expressivity, and Phase 2 consisted of applying these terms, by different observers, to the scoring of salmon expressivity as viewed in 25 video clips.

#### Phase 1 Term Generation

##### Participants

For the term generation stage, four employees of the salmon hatchery site where video recording took place, were recruited. All participants had at least 1 year of experience working directly with fish, with 3 of the 4 individuals having worked in the aquaculture industry for 7–15 years. Each observer was therefore considered experienced in monitoring fish behavior.

##### Clip Selection

For the term generation session, 12 video clips of 45 s duration were selected from footage of farmed salmon taken in December or March. Selection aimed to include a range of varied and contrasting behaviors, such as for example darting, drifting, startle responses, or interacting with artificial kelp or conspecifics. It was assumed that such footage would reflect a varied range of salmon expressions, ranging from high to low mood and arousal (39). To facilitate the generation of qualitative descriptors by observers, video clips were arranged in an order which demonstrated good expressive contrast between adjacent clips.

##### Video Viewing and Term Generation Session

The term generation session took place on-site at the salmon hatchery. Participating fish farmers were given instruction in Qualitative Behavioral Assessment and received guidance in how to generate QBA descriptors while watching the video footage. To minimize the influence of this briefing on descriptors generated by participants, any examples used referred to mammalian species and contained terms considered unlikely to be generated for fish. As the potential bank of terms was considered limited compared to other (terrestrial) species, no video practice or open discussion took place prior to the screening of the video clips. Participants were instructed to strictly refrain from any discussion during the term generation exercise.

Participants generated a total of 26 descriptive terms for salmon expression. Some of these were excluded because they described physical behavior rather than expressive demeanors (e.g., hunting, seeking), or referred to external conditions such as the fish “being controlled.”

**Figure 1:**
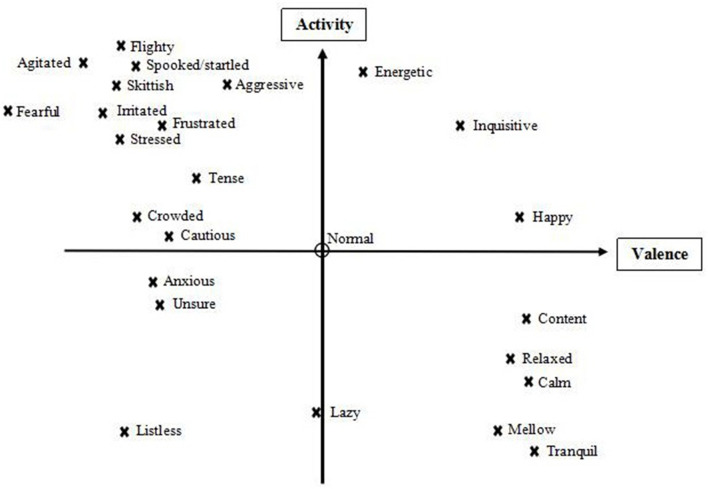
Valence and activity (arousal) scales used to discuss terms, along with placement of all generated descriptors, subject to discussion with the farmers, before finalization of the Fixed List.

After term generation was completed, participants were invited to take part in a joint discussion with the aim of selecting a final list of terms. They were asked to place all individually generated terms in a diagram in which “valence” and “activity” dimensions framed four main quadrants (39) of salmon expressivity, and to consider whether any of these quadrants were underpopulated, or whether any key terms were missing because the video footage had not shown the relevant expressions. Based on this the group added four terms: fearful, listless, frustrated and aggressive (see Figure 1). They then discussed which terms, balanced across the four quadrants, were most suitable to characterize fish body language, and chose a final list of 20 descriptors.

It was felt that the term list developed by the fish farm participants was sufficiently in line with current knowledge of fish behavior and welfare (40), and no further terms were added. This Fixed List of Descriptive Terms (Table 1) was then used in Phase 2 of QBA scoring.

**Table 1:** Finalized fixed list of QBA descriptors for salmon behavioral expression.

**Terms**			
Inquisitive	Listless	Aggressive	Mellow
Unsure	Startled	Fearful	Anxious
Agitated	Tense	Tranquil	Energetic
Relaxed	Crowded	Irritated	Stressed
Flighty	Calm	Skittish	Content

#### Phase 2: QBA Scoring Sessions

##### Participants

The 10 participants in this phase consisted of veterinary students (*n* = 4), animal welfare (MSc level) students (*n* = 5) and staff members (*n* = 1) recruited from the Dick Vet Behavior Society (University of Edinburgh). These participants had variable levels of experience in working with fish, ranging from no theoretical or practical experience (*n* = 6) to practical work in fish husbandry in a laboratory or aquarium setting (*n* = 4). No participants had experience in a commercial aquaculture setting or with salmonids.

##### Clip Selection

Twenty-five video clips of 1 min duration each were created from the on-farm footage taken in March. Clips were selected to cover as wide a range of behavioral expression in juvenile salmon as possible, and were arranged to display contrasting expressive qualities. No video clips were selected from the period 2 min before or after the feeders being deployed. Between video clips a period of 2–3 min enabled participants to record their scores for each clip on each of the 20 terms developed in phase 1.

##### Video Scoring Session

To align observers' understanding of the terms in the QBA descriptor list developed in phase 1 (Table 1), an open discussion was conducted with all participants for an hour before scoring commenced. The meaning of all 20 terms was discussed, with further time given if questions were raised against specific descriptors. Following this discussion, the term list was written out with agreed synonyms, identical in both video sessions, for each descriptor to clarify meaning (see Table 2).

**Table 2:** List of agreed synonyms for fixed list terms, as generated and discussed with all participants.

**Fixed list term**	**Agreed synonyms**
Inquisitive	Interested, curious, engaged
Unsure	Cautious
Agitated	Disturbed, unsettled
Relaxed	At ease, no urgency (not necessarily motionless)
Flighty	Erratic, volatile, unpredictable
Listless	Lethargic, lifeless
Startled	Spooked, surprised
Tense	On edge, strained
Crowded	Claustrophobic, overwhelmed
Calm	Peaceful, undisturbed
Aggressive	Hostile, assertive (violent)
Fearful	Afraid, frightened
Tranquil	Still, quiet, serene
Irritated	Annoyed, frustrated
Skittish	Excitable, easily frightened
Mellow	Easy going, tolerant, unphased
Anxious	Worried, apprehensive
Energetic	Active, lively, dynamic
Stressed	Disturbed, upset, under pressure, mix of anxious and tense
Content	Satisfied, at peace, restful

The full set of 25 video clips was then shown to all participants in two separate face-to-face sessions, for logistical reasons. Verbal instructions were given on the fundamental principles of QBA, general information on how to assess body language (using posture, gaze, speed and character of movement) and how to score descriptive terms using Visual Analog Scales as described below. These instructions were identical on both days.

Scoring was carried out on paper-based forms. For each qualitative term, a 125 mm horizontal line was present as a Visual Analog Scale. Participants were instructed to make a single vertical mark on each line, corresponding with how intensely they felt a particular expressive quality was seen in the salmon's demeanor within each clip. The leftmost extreme represents complete absence of the expressive quality (e.g., not at all agitated), and the rightmost extreme represents a maximal judgement of an expressive quality (e.g., couldn't be more agitated). Vertical marks represent where the participant has judged the clip on this spectrum. Observers were told to mark every single term for each clip, and not to use the Visual Analog Scales as a yes/no or categorized response, but to consider the whole scale when judging expressive intensity. It was explained to participants that expressive qualities of demeanor can be assessed at group level by scoring how animals collectively move; if different expressions were seen in different individual fish or different parts of a group, observers were advised to score the different expressions according to the proportion of animals showing them.

A second viewing session was required in order to collect data for intra-observer reliability analysis, i.e., the repeatability of scores within individual observers. Because it proved not possible to find a date at which all participants were available, and no further collective instruction or discussion of methods was required, the second set of video clips was transferred electronically to all participants 10 days after the first scoring event, for observation in their own home environment. The clips (*n* = 25) were identical to those used in the first video session but arranged in a different order. The observers were unaware they were the same set of clips. As before, scoring was carried out on paper-based forms using Visual Analog Scales. Participants were advised at this stage to view the video clips once only, in the given numbered order, and to allow 2–3 min for scoring after each clip. A week after delivery of the clips, paper forms were collected for data input and analysis, as carried out for the first session.

### Ethogram-Based Behavior Measurements

For the 25 video clips used for the Fixed List QBA scoring in Phase 2, an ethogram was developed consisting of categories of physical behavior that covered the different types of collective motion by fish observed in the video clips, and were sufficiently easy to visually identify to be quantified (see Table 3). These behaviors were recorded quantitatively as frequencies or durations (secs) by an independent observer who had not taken part in any of the QBA assessments.

**Table 3:** Ethogram for juvenile salmon.

**Behavior**	**Description**
“Inquisitive” (frequency)	One or more salmon are orientated toward the camera or other visible environmental features/objects, swimming in place and observing object with no erratic movements or evidence of impedance of forward motion
“Aggressive” (frequency)	One salmon is observed to make a sharp (<2 s) movement toward a conspecific which brings the aggressor into close proximity.
“Startled” (frequency)	Any number, ranging from an individual, small group to all visible fish make sudden (<2 s), sharp movements
“Calm” [duration (s)]	Consistently slow movement (2 or fewer tail movements per second), swimming in place. Can involve some drifting in position but should be passive and not associated with significant propulsive effort.
“Active” [duration (s)]	Consistent movement, smooth and not erratic. Continuous propulsion. Can vary in speed - low speed (in camera view for >3 s) and high speed (<3 s)
“Chaotic” [duration (s)]	Erratic/sharp movements in different directions with no “consensus” on direction of travel. Should involve >50% of visible group with a fast rate of travel - traverse over 1/2 of camera view in <2 s

*Behaviors were recorded as frequencies or durations as indicated in brackets*.

### Statistical Analysis of Qualitative Data

#### Measurements

On all paper forms collected from sessions 1 and 2, the distance between the vertical mark made on each completed Visual Analog Scale and the left “minimum” point was measured with a 300 mm ruler. The distance values (in millimeters) were entered into Microsoft Excel (2016), along with session and participant into a matrix formed by QBA descriptors listed horizontally in the first row, and video clip numbers listed vertically in the first column.

#### Principal Component Analysis

Principal Component Analysis (correlation matrix, no rotation) was carried out in R Studio® on the data for session 1 and session 2 separately, and also for the two sessions combined into one data set. Comparison of these 3 PCAs indicated their main dimensions of fish expressions were so similar that all further data analyses were executed with the combined data set only.

For the combined data set, Principal Components with Eigenvalues >1 were labeled by identifying the two or three highest positively and negatively loading descriptors on a Component. Where several high-loading terms were available for a Component, terms with complementary meanings were selected that together represented the larger pattern of expressivity reflected in the PCA.

Principal Component Analysis creates weighting factors allowing the scores attributed to each video clip in sessions 1 and 2 to be summarized with a numerical value on each Principal Component (the “PC score”) for each observer. These scores were the basis for the inter- and intra-observer reliability analyses of the combined data set.

#### Inter-observer Reliability

Kendall's coefficient W was used to calculate the level of agreement between the 10 participants' PC scores in the combined data set, for each of the four Principal Components. Any W values under 0.4 were considered to reflect unacceptable inter-observer reliability. This analysis was carried out using Genstat 16.1.

#### Intra-observer Reliability

The degree to which observers showed agreement between their session 1 and session 2 scores within the combined data set was determined using partial correlation, by means of a one-way ANOVA on the PC scores with either session 1 or session 2 as treatment factor. This yielded two columns of residual scores for each PC, the normality of which was evaluated by generating histogram plots and Anderson-Darling test outputs, Residual data for all 4 PCs were evaluated as normal. Pearson's correlations were then performed on these residuals for all four Principal Components. This approach ensured that data was expressed relative to the individual participants' mean score value, eliminating the influence of individual participant scoring style, ranging from conservative (limited) to full use of the VAS scales, on the results of intra-observer reliability. This analysis was carried out using Genstat 16.1.

#### Analysis of Ethogram-Based Data

The ethogram-based scores for the 25 video clips were correlated with the mean PC scores for these clips (derived by averaging each clip's session 1 and session 2 PC scores in the combined data set) on each of the 4 Principal Components. A Spearman correlation test was used as the ethogram-based scores were not normally distributed and resistant to transformation. This analysis was carried out using R Studio®.

## Results

### Qualitative Behavioral Analysis

#### Principal Component Analysis

**Figure 2:**
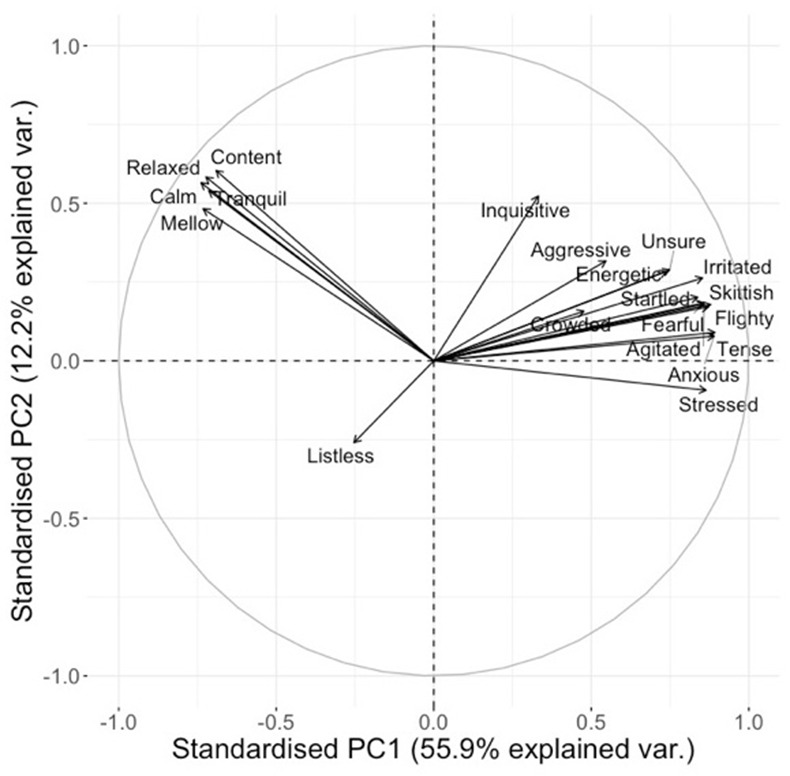
Loading plot for qualitative descriptors for the combined data set. Axes represent the level of correlation at which the QBA descriptors for fish expression load onto the two main Principal Components of the analysis.

On visual inspection of the loading plots for the separate sessions 1 and 2, and for the combined data set for both sessions, it was established that the distribution of the qualitative terms on all three plots was sufficiently similar to consider sessions 1 and 2 as representing the same “dimensions” of fish expressivity. Therefore, subsequent statistical analyses were carried out only using the combined data set for sessions 1 and 2 (Figure 2).

Four Principal Components were generated from the combined data set where the Eigen value exceeded or closely approached 1 (see Table 4) (41). Although PC4 had an Eigen value of slightly <1, it was considered to reflect an interesting dimension of fish behavioral expression, and was therefore included. The first dimension (PC1) explained the greatest percentage of variance at 56%, with the first four dimensions collectively accounting for 79% of the variation (see Table 4).

**Table 4:** Eigen values and percentage of variance for each principal component.

**Value**	**PC1**	**PC2**	**PC3**	**PC4**
Eigen value	**11.17**	**2.441**	**1.275**	0.910
% of variance explained	56	12	6	5
Cumulative variance	56	68	74	79

*Eigen values greater than 1 are in bold*.

As can be seen in Table 5, PC1 ranges from Tense/anxious/skittish to Calm/mellow/relaxed, describing a shift from negative mood/high-energy to positive mood/low-energy. PC2 ranges from Content/relaxed to Listless, but as “Listless” is the only significantly negatively loading descriptor, this dimension seems to mainly reflect the salmons' degree of listlessness against all other possible expressions. PC3 ranges from Listless/crowded to Energetic/inquisitive, and appears to indicate an association between listlessness and crowded conditions. PC4 ranges from Inquisitive/crowded to Fearful/flighty and seems to indicate a contrast between inquisitiveness and fear.

**Table 5:** Descriptor loading values for each principal component.

**Term**	**PC1**	**PC2**	**PC3**	**PC4**
Inquisitive	0.099	0.335	**−0.164**	**−0.609**
Unsure	0.224	0.185	0.034	0.022
Agitated	0.257	0.116	**–**0.000	0.133
Relaxed	**−0.216**	**0.374**	0.039	0.090
Flighty	0.261	0.110	−0.004	**0.213**
Listless	−0.076	–**0.166**	**0.729**	0.092
Startled	0.251	0.129	−0.004	0.205
Tense	**0.267**	0.058	0.147	−0.024
Crowded	0.143	0.101	**0.431**	–**0.516**
Calm	–**0.221**	**0.362**	0.044	0.053
Aggressive	0.164	0.203	0.180	–**0.227**
Fearful	0.256	0.118	0.043	**0.223**
Tranquil	−0.214	0.346	0.191	0.184
Irritated	0.255	0.168	0.109	0.012
Skittish	**0.263**	0.114	−0.013	0.197
Mellow	–**0.219**	0.309	0.185	0.185
Anxious	**0.266**	0.050	0.065	0.137
Energetic	0.221	0.182	–**0.297**	−0.021
Stressed	0.259	−0.060	0.112	0.076
Content	−0.207	**0.387**	−0.094	0.068

*The highest positively and negatively loaded terms for each PC are in bold*.

#### Inter-observer Reliability

PC1 (Tense/anxious/skittish - Calm/mellow/relaxed) was the only Principal Component to demonstrate good inter-observer reliability for the combined data set (*W* = 0.68, χ^2^ = 335.31, *P* = < 0.001). The other 3 PCs had W values of below 0.4 for all data sets, which is considered unacceptable (42).

#### Intra-observer Reliability

Similarly Tense/anxious/skittish -Calm/mellow/relaxed (PC1) was the only dimension to show good intra-observer reliability between session one and session two PC scores in the combined data set (PC1: *r* = 0.65, p < 0.001), with PC2-PC4 demonstrating significant but poor to moderate correlations.

### The Association Between QBA Scores and Ethogram-Based Behavior Measurements

Given that PC1 was the only QBA dimension with significant inter- and intra-observer reliability, here we report only significant correlations between ethogram-based behaviors and PC1 that were of sufficient strength (i.e., *r* > ± 0.50).

**Figure 3:**
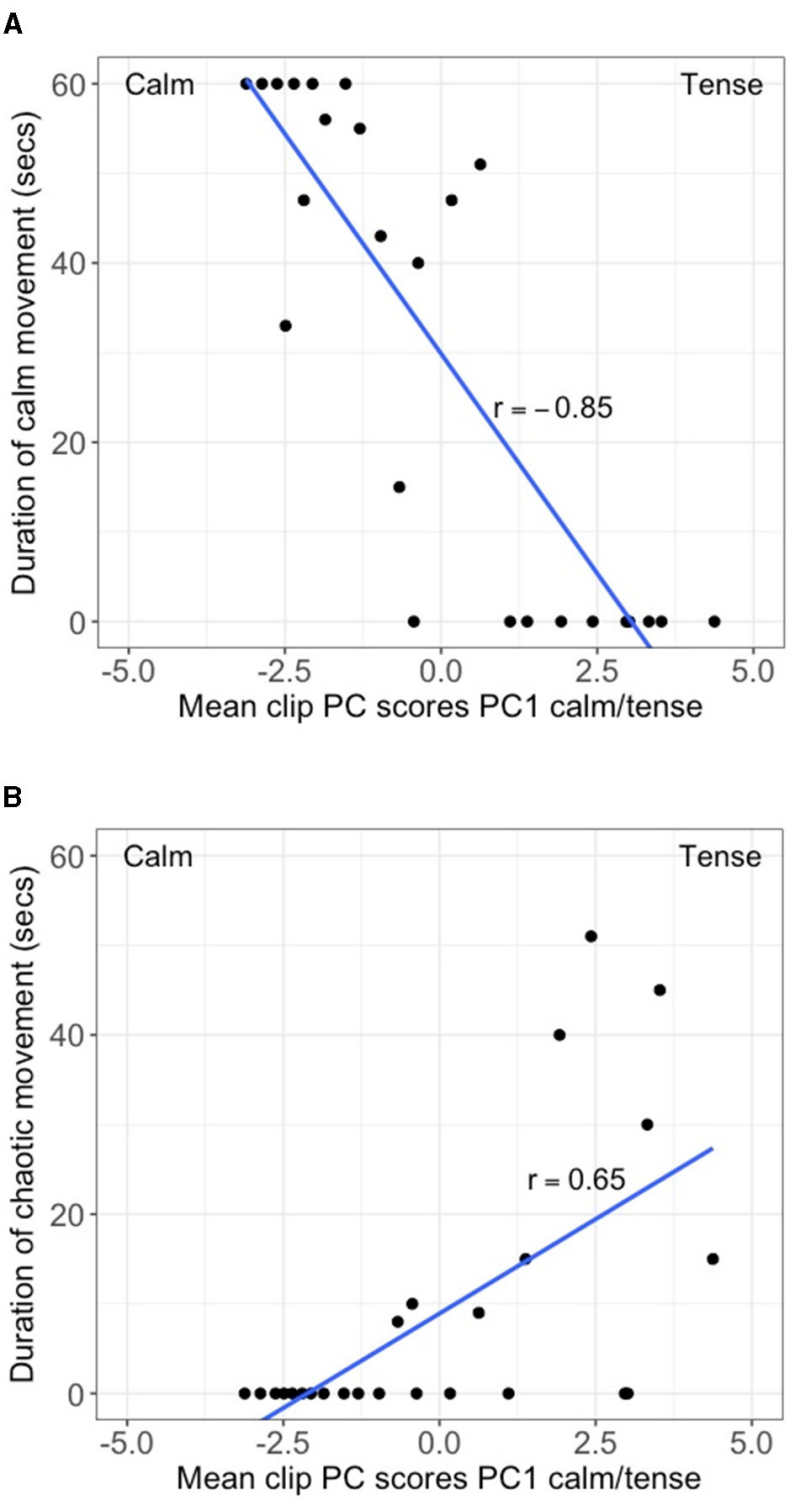
Scatterplot of mean clip PC scores for PC1 (Tense/Calm) vs. **(A)** duration of calm movement in seconds, and **(B)** duration of chaotic movement in seconds. Line of best fit is included.

PC1 scores (Tense/anxious/skittish – Calm/mellow/relaxed) showed a very strong negative correlation (*r* = −0.85, *p* =< 0.001) with the duration of slow physical movement without active propulsion (Figure 3A). In addition PC1 scores showed a moderate positive relationship (*r* = 0.65, *p* =< 0.01) with the duration of erratic/sharp movement in different directions (Figure 3B). Correlations between PC1 scores and other ethogram-based measures were non-significant.

## Discussion

The aim of this study was to investigate the potential of QBA as a welfare assessment tool for Atlantic Salmon in the freshwater phase within aquaculture. In a first phase of the study, experienced fish famers watched a set of 12 videos created to cover a wide range of behavioral expression in juvenile salmon, and through discussion created a list of 20 descriptors for salmon expressivity. In a second phase, this fixed list of terms was used by 10 different observers, all inexperienced in fish farming, to score salmon expressivity from a new set of 25 video clips. In order to test the repeatability of their scores, these observers scored the same 25 clips a second time at least 10 days after the first session had been completed. The initial session was conducted face to face with all assessors together, but the repeat scoring of clips was performed online by participants individually at their own home. The ensuing data were all analyzed together using Principal Component Analysis, revealing four meaningful dimensions of salmon expression. However, of these dimensions only the first one (PC1) showed acceptable inter- and intra-observer reliability.

PC1 was characterized as ranging from “Tense/anxious/skittish” to “Calm/mellow/relaxed,” explaining 56% of the variation, and showing good inter- and intra-observer reliability (the latter despite the different settings in which the two sessions were conducted). Significant correlations with measures of the salmons' physical movements found “Calm/mellow/relaxed” demeanor to be associated with slow, unpropelled movement, and “Tense/anxious/skittish” demeanor with erratic/sharp movement in different directions. Such meaningful mapping of qualitative assessments and ethogram-based measurements supports the validity of QBA (43), however we should not necessarily expect a full overlap of the two types of assessment. Qualitative assessments include and integrate subtle expressive aspects of an animal's demeanor in its environmental and social context, that may be difficult to quantify in ethogram-based categories. Thus, QBA is hypothesized to provide information on an animal's affective state that is complimentary to other measures and facilitates a more comprehensive evaluation of animal welfare, including positive welfare states (25). The current results indicate that observers with relatively little fish-based experience (but using descriptors developed by experienced fish farmers) were able to consistently judge a dimension of tense vs. calm expressivity in juvenile salmon, which is highly relevant to appraisals of fish welfare in aquaculture.

The remaining dimensions (PCs 2, 3, and 4) explained a lower percentage of variation, and did not show acceptable inter- and intra-observer reliability. A first reason for this may be that the expressions characterizing these dimensions (e.g., listless, content, inquisitive) were more difficult to perceive and assess for inexperienced observers than those characterizing PC1, particularly as the latter were associated with specific patterns of physical movement. “Listless” appeared as a key term in characterizing both PC2 and PC3, but for inexperienced observers it will not have been easy to distinguish “calm/relaxed” fish from “listless” fish. A contributing factor here may have been the large numbers of fish shown in the video clips, making it harder to clearly see expressive cues. In other QBA studies, video footage is often focused on individual animals [e.g., (44, 45)], or is focused on small groups of animals (<15) with low stocking density [e.g., (43)]. However, QBA methodology has also been successfully applied to larger groups of terrestrial animals, for example through the EU Welfare Quality® assessment protocols [e.g., (29)], or in studies of farmed broiler chickens (37). In order for QBA to be successful as an on-farm welfare assessment tool within aquaculture it has to be robust when observing very large groups of fish. This study provides the first evidence for the availability of a meaningful and reliable dimension of salmon expressivity, describing the difference between “Tense/anxious/skittish” and “Calm/mellow/relaxed” fish.

Similar dimensions have been reported for terrestrial animals, such as in cattle during transport (46), or at the abattoir (47). Many QBA studies of terrestrial animals find main dimensions of mood and energy that show acceptable inter-observer agreement and can be applied to practical farm-assessment, however as the single reliable dimension identified here characterizes a combined shift in both mood and energy, it could still be of use for practical application to welfare assessment in farmed salmon. In fact all four dimensions identified in the current study describe combined shifts in mood and energy, and so are potentially relevant to monitoring welfare in farmed fish. The question is whether further study could improve inter-observer agreement for these dimensions, through a stronger focus on the experience and training required for observers. Considering this study's observers' experience, a second reason for not finding more reliable dimensions could be that observers struggled to apply unfamiliar descriptors to an unfamiliar species. In this study we invited an experienced group of fish farmers to create the list of QBA descriptors, rather than asking the observers in phase 2 to create their own descriptors for scoring [e.g., (48)]. The reason for this was that QBA term lists should cover a comprehensive and varied range of a species' expressions, and that there is considerable risk that inexperienced observers will fail to include important aspects of this expressivity (49). The potential downside of this approach, however, is that prescribing a list of terms which observers have not developed themselves makes it harder for them to use it appropriately (31). Observers were asked to spend an hour discussing the meaning of the terms on the list. However, in the absence of much experience with farmed fish, this may not have been sufficient to reach agreement on dimensions beyond the first obvious one (50). On the other hand, some studies have reported that non-experienced observers show better agreement than experienced ones, arguing that factors such as observer personality and attitude are more powerful determinants of agreement than experience (51). Thus, regardless of observers' levels of experience, the use of pre-fixed QBA term lists in fish requires that adequate instruction and training in fish biology and behavior is provided (50, 52). The consequence of this for QBA's feasibility is that initially it may require considerable investment in observers' assessment skills. However, this is true for most assessment methods (31), and the investment should pay off over time in creating an efficient and informative assessment tool.

Further QBA research should extend to assessment of salmon at different lifecycle phases and in different production environments. It would be informative to combine use of QBA with other welfare assessment systems for salmon, such as for example reviewed by Stien et al. (23). In addition it could be fruitful to combine QBA with other monitoring modalities such as motion-detection of optical flow at group level, as studied in poultry by Dawkins et al. (53). This could be taken forward into remote sensing and machine learning, using QBA to evaluate the capacity of such new technologies to address valence aspects of animal welfare. QBA in its own right, when applied by trained farm staff assisted by mobile application technology, also has considerable potential as an on-farm welfare assessment tool within aquaculture, as it is observational, non-invasive, and, after initial investment in observer training, can be applied in a time efficient manner (26). Using all such development avenues, QBA descriptor lists and dimensions can be further validated and tested for efficacy. Given the growth of aquaculture globally and the large number of species and individual fish involved (4), there is a need to develop reliable tools for monitoring fish welfare (including positive welfare), that can inform guidance and legislation to meet the species-specific needs of fish.

## Conclusion

This study provides the first successful application of QBA to the assessment of emotional expressivity in Atlantic salmon under farmed conditions. One dimension (Tense/anxious/skittish – Calm/mellow/relaxed) explained the majority of variation, showed good inter- and intra-observer reliability, and correlated significantly to durations of erratic vs. slow physical movement. Thus, QBA has the potential to provide a meaningful and, with further validation, time-efficient tool for welfare assessment in farmed salmon.

## Data Availability Statement

The datasets presented in this study can be found in online repositories. The names of the repository/repositories and accession number(s) can be found at: https://hdl.handle.net/10283/3902.

## Ethics Statement

The studies involving human participants were reviewed and approved by University of Edinburgh Vet School Human Ethical Research Committee. The patients/participants provided their written informed consent to participate in this study. The animal study was reviewed and approved by University of Stirling Animal Welfare & Ethical Review Body.

## Author Contributions

The collection of video footage was conducted by ME. The term generation and QBA sessions were conducted by ME and Laura Dunn under supervision of SJ and FW. SR and JT were also supervisors of ME's PhD project and were involved in experimental design and preparation of the manuscript. Statistical analysis was carried out by ME and Laura Dunn under supervision of SJ, FW, and SR. Preparation of the manuscript was conducted by SJ with input from all authors.

## Funding

This study reported in this paper was part of ME's PhD project at the Institute of Aquaculture at Stirling University, which was co-funded by SRUC and the University of Stirling. It was also part of the MSc project of Laura Dunn for the MSc in Applied Animal Behavior and Animal Welfare Science at the University of Edinburgh. The Rural & Environmental Science & Analytical Services Division (RESAS) of the Scottish Government provided additional funding supporting QBA research at SRUC.

## Conflict of Interest

The authors declare that the research was conducted in the absence of any commercial or financial relationships that could be construed as a potential conflict of interest. The handling editor declared a past co-authorship with one of the authors SR.

## Publisher's Note

All claims expressed in this article are solely those of the authors and do not necessarily represent those of their affiliated organizations, or those of the publisher, the editors and the reviewers. Any product that may be evaluated in this article, or claim that may be made by its manufacturer, is not guaranteed or endorsed by the publisher.

## Footnotes

^1^Council Directive 98/58/EC of 20 July 1998 Concerning the Protection of Animals Kept for Farming Purposes. Official Journal of the European Communities. L221/23-27. https://www.legislation.gov.uk/eudr/1998/58/pdfs/eudr_19980058_adopted_en.pdf

^2^Treaty of Lisbon Amending the Treaty on European Union and the Treaty Establishing the European Community 2007/C 306/01 of 13 December 2007.

^3^Council Directive 2010/63/EU of 20 of October 2010 on the protection of animals used for scientific purposes. Official Journal of the European Union. L 276/33-79. https://eur-lex.europa.eu/LexUriServ/LexUriServ.do?uri=OJ:L:2010:276:0033:0079:en:PDF

^4^Animals (Scientific Procedures) Act 1986 Legislation. The National Archives. https://www.legislation.gov.uk/ukpga/1986/14/contents
